# The role of composition of gut microbiota in reflecting the severity of acute pancreatitis is probably overstated

**DOI:** 10.1128/spectrum.00184-24

**Published:** 2024-04-08

**Authors:** Jia-Lin Wu, Jun-Yang Luo, Xin-Yi Deng, Tsz Yu Lau, Zai-Bo Jiang

**Affiliations:** 1 Department of Interventional Radiology, The Third Affiliated Hospital of Sun Yat-sen University, Guangzhou, China; 2 Zhongshan School of Medicine, Sun Yat-sen University, Guangzhou, China; Institut National de Santé Publique du Québec, Sainte-Anne-de-Bellevue, Québec, Canada

**Keywords:** gut microbiota, acute pancreatitis, Mendelian randomization

## LETTER

We read Wang et al.’s original manuscript with great interest (1). The authors demonstrated that alterations and functional profiles of the microbiome can reflect the severity and etiology of acute pancreatitis (AP). While the study was elegantly designed, the association between gut microbiota and AP still has some issues that merit further discussion. Mendelian randomization (MR) design is a type of instrumental variables (IVs) analysis, which uses genetic variants as the IVs to estimate the association between risk factors and disease outcomes (2). MR design can overcome the limitations of observational studies, as genetic variants, specifically single-nucleotide polymorphisms (SNPs), are randomly allocated at conception (3). Herein, we attempted to explore whether the gut microbiota is causally associated with APs through a two-sample MR analysis.

Summary statistics from a publicly available genome-wide association study (GWAS) meta-analysis, including a total of 18,340 individuals, were utilized for gut microbiota (4). The GWAS summary statistics for AP were obtained from FinnGen consortium R10 release data (6,787 cases and 361,641 controls) (5). Details regarding ethical approval can be found in the original research. SNPs robustly related to gut microbiota at a GWAS threshold of statistical significance (*P* < 5 × 10^−8^, LD *r*^2^<0.001) were selected as IVs. Inverse-variance weighted (IVW) was used as the main MR analysis method. Besides, sensitivity analysis was tested by weighted media, MR Egger, and MR-PRESSO. All statistical analyses were performed in R software (Version 4.2.3).

After conducting a series of quality control and sensitivity analysis, the IVW method suggested that genetic liability to the gut microbiota was not causally associated with APs after the Bonferroni test. In a previous study, SNPs smaller than the strict threshold (*P* < 1 × 10^−5^) were selected as the IVs to identify potential causal associations. However, no discernible genetic liability to the gut microbiota was found to be causally associated with APs, as per the Bonferroni test (6).

Explicitly, the conclusion drawn in these two studies contradicts the findings of the authors. Several potential explanations for this disparity can be identified. First, the previously observed associations of gut microbiota with AP may have been influenced by confounding factors, including gender, previous medication and dosages, and pre-existing diseases. Second, Wang et al. primarily concentrated on 13 mild AP cases and only 7 patients with severe AP; therefore, the relatively limited sample size of AP cases may have impacted the overall findings. Third, smoking has been found associated with an increased risk of several gastrointestinal diseases, and a previous MR study demonstrated that genetic predisposition to smoking was associated with AP (7). Thus, all these factors may have impacted the conclusions regarding the risk of AP.

In summary, the current study revealed that there was no causal link from gut microbiome to AP, suggesting the observed relationship in the previous study may be biased. In future research, the integration of multiple omics platforms and the encouragement of causal investigations with larger sample sizes are crucial for advancing our understanding of disease pathogenesis and etiology.

## EDITOR'S NOTE

**Ed. Note**: The authors of the original article did not respond at the time of publication.

## References

[1] Wang Z, Guo M, Li J, Jiang C, Yang S, Zheng S, Li M, Ai X, Xu X, Zhang W, He X, Wang Y, Chen Y. 2023. Composition and functional profiles of gut microbiota reflect the treatment stage, severity, and etiology of acute Pancreatitis. Microbiol Spectr 11:e0082923. doi:10.1128/spectrum.00829-23 37698429 PMC10580821

[2] Smith GD, Ebrahim S. 2003. 'Mendelian randomization': can genetic epidemiology contribute to understanding environmental determinants of disease? Int J Epidemiol 32:1–22. doi:10.1093/ije/dyg070 12689998

[3] Emdin CA, Khera AV, Kathiresan S. 2017. Mendelian randomization. JAMA 318:1925–1926. doi:10.1001/jama.2017.17219 29164242

[4] Kurilshikov A, Medina-Gomez C, Bacigalupe R, Radjabzadeh D, Wang J, Demirkan A, Le Roy CI, Raygoza Garay JA, Finnicum CT, Liu X, et al.. 2021. Large-scale association analyses identify host factors influencing human gut microbiome composition. Nat Genet 53:156–165. doi:10.1038/s41588-020-00763-1 33462485 PMC8515199

[5] Kurki MI, Karjalainen J, Palta P, Sipilä TP, Kristiansson K, Donner KM, Reeve MP, Laivuori H, Aavikko M, Kaunisto MA, et al.. 2023. FinnGen provides genetic insights from a well-phenotyped isolated population. Nature 613:508–518. doi:10.1038/s41586-022-05473-8 36653562 PMC9849126

[6] Wang K, Qin X, Ran T, Pan Y, Hong Y, Wang J, Zhang X, Shen X, Liu C, Lu X, Chen Y, Bai Y, Zhang Y, Zhou C, Zou D. 2023. Causal link between gut microbiota and four types of pancreatitis: a genetic association and bidirectional Mendelian randomization study. Front Microbiol 14:1290202. doi:10.3389/fmicb.2023.1290202 38075894 PMC10702359

[7] Yuan S, Chen J, Ruan X, Sun Y, Zhang K, Wang X, Li X, Gill D, Burgess S, Giovannucci E, Larsson SC. 2023. Smoking, alcohol consumption, and 24 gastrointestinal diseases: Mendelian randomization analysis. Elife 12:e84051. doi:10.7554/eLife.84051 36727839 PMC10017103

